# A New Approach to Harness Probiotics Against Common Bacterial Skin Pathogens: Towards Living Antimicrobials

**DOI:** 10.1007/s12602-021-09783-7

**Published:** 2021-04-15

**Authors:** Ghazi Khalfallah, Rita Gartzen, Martin Möller, Elisabeth Heine, Rudolf Lütticken

**Affiliations:** 1 DWI Leibniz-Institute for Interactive Materials, 52074 Aachen, Germany; 2 RWTH Aachen University, Aachen, Germany; 3 Institute of Technical and Macromolecular Chemistry, RWTH Aachen University, 52074 Aachen, Germany

**Keywords:** Beneficial bacteria, *Lactobacillales*, *Lactobacillus plantarum*, *Lactiplantibacillus plantarum*, Skin disorder, Acne vulgaris, Treatment

## Abstract

In this study, the potential of certain lactic acid bacteria—classified as probiotics and known to be antimicrobially active against pathogens or food-poisoning microorganisms—was evaluated with respect to their activity against bacterial skin pathogens. The aim of the study was to develop a plaster/bandage for the application of inhibitory substances produced by these probiotics when applied to diseased skin. For this purpose, two *Streptococcus salivarius* strains and one *Lactobacillus plantarum* were tested for production of antimicrobials (bacteriocin-like substances) active against Gram-positive and Gram-negative pathogens using established methods. A newly designed membrane test ensured that the probiotics produce antimicrobials diffusible through membranes. Target organisms used were *Cutibacterium acnes*, *Staphylococcus aureus*, and *Pseudomonas aeruginosa*. Moreover, the *L. plantarum* 8P-A3 strain was tested against additional bacteria involved in skin disorders. The *Lactobacillales* used were active against all potential skin pathogens tested. These probiotics could be enclosed between polymer membranes—one tight, the other permeable for their products, preserved by vacuum drying, and reactivated after at least three months storage. Importantly, the reactivated pads containing the probiotics demonstrated antibacterial activity on agar plates against all pathogens tested. This suggests that the probiotic containing pads may be topically applied for the treatment of skin disorders without the need for a regular antibiotic treatment or as an adjunctive therapy.

**Supplementary Information:**

The online version contains supplementary material available at 10.1007/s12602-021-09783-7.

## Introduction

The worldwide rising problem of antibiotic resistance in bacterial pathogens [1, 2] calls for searches for alternative and/or adjunctive ways of antimicrobial therapy [3–7]. In addition to the selective pressure any use of antibiotics exerts, there are other restrictions for the application of antibiotics such as allergy against many groups of antibiotics [8]. There are also reports that non-antibiotic antiseptics, topically applied to diseased skin and mucous membranes or to infected wounds, might be less active due to development of resistances; those might also pose problems with allergic reactions [9].

Considering this, the employment of probiotic bacteria producing bacteriocin-like substances for topical application onto the skin to treat skin disorders associated with bacterial pathogens might be an alternative to the topical or systemic use of antibiotics or the application of antiseptics [10, 11]. We are using the term “probiotic” here—in the sense of “beneficial bacteria”—since the lactic acid bacteria (LAB) applied were originally employed by the oral route. Probiotics from different bacterial taxonomic units have been described to be applied for treatment or prevention of diseases directly onto surfaces of the human body, e.g., the oral cavity or the skin. Examples for this are the application of *Streptococcus salivarius* subsp. *salivarius* (commonly abbreviated as *S. salivarius*) strain K12 or *S. salivarius* M18 for the prevention of sore throat caused by *Streptococcus pyogenes* and the prevention of pneumococcal otitis media, or for prophylaxis of dental caries, respectively [12–16]. Also, other probiotics are considered or already on the market to be applied directly onto the skin for cosmetic or medical treatment purposes [17, 18]. Extracts from probiotic bacteria were also discussed to be used for skin applications [19–22].

Skin disorders which are treated with antibiotics (among other therapeutic measures) include acne (*Acne vulgaris*), infected atopic eczema lesions, venous leg ulcers, and (burn) wound infections [23–26]. The respective pathogens associated with these diseases comprise the Gram-positive bacteria *Cutibacterium acnes* (formerly *Propionibacterium acnes*) and *Staphylococcus aureus* [27]*,* and Gram-negative rods like *Pseudomonas aeruginosa, Enterobacterales* (*E. coli*, *Klebsiella* spp. etc.), and others [28].

Consequently, we studied the in vitro activity of selected probiotic *Lactobacillales* strains against those common skin pathogens, including antibiotic resistant strains. The probiotics were applied in a unique way by enclosing them between membranes, thereby allowing their products to diffuse onto surfaces inoculated with the pathogens. This should serve as a model for a “probiotic pad” (bandage, plaster, patch) to be applied for the treatment of various skin disorders.

Strains of *S. salivarius* and *Lactobacillus plantarum*—for the sake of convenience, we use the former nomenclature for *Lactobacillus* species instead of the recent changes [29] in this publication—were extensively studied in recent years either as probiotics conferring health benefits to the host or as natural food preservatives, and their efficiency and safety have been proved [12–16, 30–36]. Bacteria produce a wide range of inhibitory substances: classical low-molecular weight antibiotics, metabolic products, lytic agents, enzymes, bacteriocins, and “defective prophages” [37]. LAB are known to be producers of metabolic products exerting antimicrobial activity: organic acids, especially lactic acid (giving the name as LAB to this vast group of bacteria), hydrogen peroxide, and diacetyl. The LAB *S. salivarius* K12, *S. salivarius* M18, and *L. plantarum* 8P-A3 used here are additionally able to secrete small ribosomally synthesized antimicrobial peptides (bacteriocins), a feature increasing their antagonistic activity against other bacteria as described previously and deduced from comparative genomic data [38–43].

The increasing concerns over uncritical antibiotic treatments of skin disorders, in particular *Acne vulgaris* [23], and of wound infections have led to considerations of the use of alternative treatment methods, in particular the application of probiotic bacteria or their antimicrobial products. Although the spectrum of bacteriocins produced by probiotic bacteria is usually narrow and restricted to close relatives of the producers, there are exceptions described in the literature with probiotic LAB having broader spectra including common pathogens [41, 44, 45].

We decided for this novel approach to apply the probiotic bacteria not directly onto the skin but enclosed between membranes for the following reasons: Although bacteria used as orally administered probiotics are generally regarded as safe (GRAS-status, in some countries approved as such), their direct application onto diseased skin may involve a residual risk, especially in immunocompromised patients or patients with unknown immune status. It is well known that some *Lactobacillales*, in particular “viridans” streptococcal species, are common causes of sepsis in immunosuppressed patients [46–48].

The concept of enclosing microorganism between membranes and switching off and on their metabolism by diffusion of nutrients and water and thereby delivering metabolites to the outside environment had been proven with the fungus *Penicillium roqueforti* [49]. We decided to use living beneficial bacteria and their antimicrobial potential instead of purified bacteriocins, thereof, because these bacteria are often able to produce several antimicrobially active substances simultaneously, depending on their growth cycle and quorum-sensing machinery. In this approach, environmental stimuli such as external inducers for bacteriocin production [50, 51]—either from skin bacterial flora or from skin cells—may diffuse into the enclosure thus enhancing the antimicrobial product yield.

## Materials and Methods

### Bacterial Strains, Culture Media, and Growth Conditions

The probiotic bacterial strains used in this study were obtained from the sources listed in Table 1; they were maintained in “cryotubes” (CRYOINSTANT Mixed, pH 7.3 ± 0.2, VWR International GmbH, Darmstadt, Germany) and stored at a temperature below −20 °C.

**Table 1:** Probiotic bacteria evaluated for antagonistic activity

Strain	Origin	Isolated from	References
*Lactobacillus plantarum* 8P-A3	Alexander Suvorov, Institute of Experimental Medicine, Dept. Molecular Microbiology, St. Petersburg, Russia	“Lactobacterinum siccum” (Microgen, Moscow, Russia)	[41]
*Streptococcus salivarius* K12	BLIS K12™ probiotic powder, provided by Prof. John R. Tagg, University of Otago, Dunedin, New Zealand	Saliva of a healthy child	[52, 53]
*Streptococcus salivarius* M18	ProBio-Dent® lozenge for teeth and mouth care (Syxyl GmbH & Co. KG, Cologne, Germany)	Healthy mouth flora	[12, 43, 54]

The target bacterial strains tested against the three probiotics, applying the antagonism test methods, are listed in Table 2.

**Table 2:** Pathogenic bacteria tested for sensitivity to the probiotics

Cell wall structure	Bacterial strain	Received from collection	Origin	Comment
Gram positive	*Staphylococcus aureus* subsp. *aureus* DSM-799/ ATCC 6538	ATCC	Human lesion	Standard strain in use for disinfectant testing
	*Cutibacterium acnes* DSM-1897/ ATCC 6919	DSMZ (German Collection of Microorganisms and Cell Cultures GmbH)	Acne lesion in human facial skin	Quality control strain
Gram negative	*Pseudomonas aeruginosa* DSM-1117/ ATCC 27,853	DSMZ	Human, blood culture	Quality control strain for antibiotic sensitivity testing

For convenience, all *Staphylococcus aureus* subsp. *aureus* strains used were termed *Staphylococcus aureus* (*S. aureus*) in the text.

The following culture media were used throughout the study: Brain–heart infusion (BHI) (Carl Roth GmbH & Co. KG, Karlsruhe, Germany), De Man-Rogosa-Sharpe (MRS) broth (Carl Roth), Standard Nutrient Agar I (ST I, Merck KGaA, Darmstadt, Germany), and Wilkins-Chalgren anaerobe broth (WC, OXOID, ThermoFisher Scientific, Thermo Fisher Scientific, Waltham, MA, USA). For solid media, 1.2% agar (Agar–Agar, bacteriological, Carl Roth) was added to the respective broth media. Incubation was performed as described below; for anaerobic cultures, GasPak™ EZ incubation systems (Becton, Dickinson and Company – BD Diagnostic Systems, Franklin Lakes, NJ, USA) were used. Incubation temperature for bacterial cultures used was 33 °C in most cases (as a compromise between skin temperatures at different sites and the temperature optimum of the test bacteria) if not stated otherwise and based on the original description of the method applied. Bacterial cell counts (colony forming units, CFU) were determined by the plate count method as appropriate.

### Antagonism Tests

#### Line Test

The test was performed according to Moore et al. [55] with modifications. Fifteen microliter of *P. aeruginosa*, *S. aureus*, or *C. acnes* (Table 2) cell suspension diluted to 10^5^ CFU/mL were first applied onto BHI agar medium (at the edge of the plate); then, the plate was held at an angle so that the liquid could slowly flow downwards. After drying of the pathogen suspension, 15 µL of the probiotic was applied in two dilutions from the edge of the plate; then, the plate was held at an angle so that the liquid could slowly flow downwards at a right angle to the pathogen streak. Both probiotic dilutions were not applied simultaneously to ensure the retention of straight, parallel lines, perpendicular to the pathogen streak. The second probiotic dilution was applied after drying of the first dilution. *S. salivarius* M18 was inoculated with 2 × 10^4^ and 2 × 10^5^ CFU/mL, *L. plantarum* 8P-A3 with 3 × 10^7^ and 3 × 10^9^ CFU/mL, and *S. salivarius* K12 with 10^6^ and 10^7^ CFU/mL, respectively; these numbers had proved as most suitable for the line tests in our hands. Agar plates inoculated with the pathogens *S. aureus* and *P. aeruginosa* were first incubated anaerobically at 33 °C for 48 h to allow sufficient growth of the probiotic, then aerobically for 24 h at the same temperature. The test plates with *C. acnes* were incubated only anaerobically at 33 °C for 5 to 7 days.

#### Double Layer Agar Test

This method was performed essentially as described previously by Tsapieva et al. [41]. It comprises the incorporation of the producer strain into the lower agar medium layer with a final cell count of 10^5^ CFU/mL. *L. plantarum* 8P-A3 was incorporated into MRS agar (lower layer) and *S. salivarius* M18 and K12 into BHI and WC agar, respectively. After this medium had solidified, a medium suitable for the target bacteria (ST I for *P. aeruginosa* and *S. aureus*, BHI for *C. acnes*) was poured onto the first layer. The pathogenic target bacteria were inoculated onto the upper layer in three dilutions and in triplicate. As a control, plates without probiotic bacteria in the lower layer were employed. In the tests with *S. salivarius* M18 as producer strain, *S. aureus* and *P. aeruginosa* were diluted to 10^4^, 5 × 10^3^, and 2 × 10^3^ CFU/mL resp. and the plates were incubated aerobically at 33 °C for 24 h. *C. acnes* was diluted to the same density as the other pathogens but the plates were incubated anaerobically for seven days at 33 °C. *P. aeruginosa* and *S. aureus* were diluted to 10^7^, 10^8^, and 10^9^ CFU/mL when tested for sensitivity to *S. salivarius* K12 and *L. plantarum* 8P-A3. These plates were incubated aerobically at 33 °C for 3 to 5 days.

#### Membrane Test 

In this method, the target bacteria were first incorporated into the agar medium with a final density of 10^3^ CFU/mL. After solidification of the medium, cellulose acetate membranes (Sartorius Stedim Biotech GmbH, Göttingen, Germany) were applied onto the agar surface. Different inocula of the probiotics were pipetted onto the membranes; then, the plates were incubated anaerobically at 33 °C for up to 7 days. Tests with *S. salivarius* M18: The pathogens *S. aureus* and *C. acnes* were incorporated into BHI and WC agar, respectively. This probiotic was applied onto the membrane in suspension of 2 × 10^6^ CFU/mL and tenfold concentrated. Tests with *S. salivarius* K12: 10^8^ CFU/mL were applied onto the membrane. In all the tests, the liquid culture medium was used as control. The volume of each probiotic suspension or culture medium (control) applied onto the membrane spots was 20 µL.

#### Deferred Antagonism Test

This method was performed essentially as described previously [38, 56]. After overnight culture (*S. salivarius* in WC broth with 0.1% (v/v) Tween 80, *L. plantarum* in MRS broth), 15 µL of *S. salivarius* M18 (2 × 10^6^ CFU/mL) or *L. plantarum* 8P-A3 (3 × 10^9^ CFU/mL) were pipetted onto the surface of Columbia agar with 5% sheep blood, then drained down the agar plate as described for the line test. The plates were incubated anaerobically at 37 °C for 18 h. The next day, the probiotics were removed from the agar medium using a sterile cotton swab, and then the residual probiotics on the plates were killed by incubation for 30 min upside-down over filter paper soaked with chloroform. The plates were then aerated for another 30 min to remove the residual chloroform. *C. acnes* cell suspension (15 µL, 10^5^ CFU/mL) was applied at a right angle across the producer streak, and the plates were incubated anaerobically at 37 °C for 5 days. For this test, two control plates were used, where the pathogen and the probiotic were cultivated separately (Control 1: only a pathogen streak, control 2: only a probiotic streak). Tests to detect a deferred antagonism against *S. aureus* and *P. aeruginosa* were performed accordingly.

#### Fabrication of a Laboratory Prototype Pad Enclosing Probiotic Bacteria

A polycarbonate membrane, impermeable for bacteria (Type: Makrofol^®^ N, RCT^®^-GDF-CT, thickness: 0.02 mm, Reichelt Chemietechnik GmbH + Co, Heidelberg, Germany) and a second, semipermeable (Type: Nucleopore^®^, pore size: 0.2 µm, diameter: 50 mm, Whatman, supplied by Reichelt Chemietechnik) were heat-sealed using a bag sealer (Fermant 22 N-R, joke Folienschweisstechnik GmbH, Bergisch Gladbach, Germany) at 170 °C, for 8 s at three margins. 2 cm × 2 cm Viscose/polypropylene nonwoven (M1556, Freudenberg SE, Weinheim, Germany) was inserted between the sealed polymer membranes as a cell carrier material, then heat-sterilized by autoclaving for 20 min at 121 °C. The probiotics *S. salivarius* K12 and *L. plantarum* 8P-A3 were subsequently applied to the insert within the pouch as a suspension. The same culture medium used for the initial cultivation of the probiotics was used as suspension medium except for the pouches filled with *L. plantarum* to be tested against *C. acnes*, since this pathogen is sensitive to the acidic pH of the MRS broth. In this case, the suspension medium was phosphate-buffered saline (PBS). The suspension of *L. plantarum* 8P-A3 was adjusted so that it contained 3–4 × 10^10^ probiotic bacteria per mL and 5% trehalose (m/v) as protectant. The suspension of *S. salivarius* K12 or M18 contained 1–4 × 10^8^ and 1–2 × 10^7^ CFU/mL, respectively, and the same amount of trehalose as for *L. plantarum* 8P-A3. 250 µL from each cell suspension were applied inside the pads. As negative controls, solutions containing only the suspension medium (culture medium or PBS) and the protectant were used. The probiotic pouches were then equilibrated at 4 °C for at least 15 min and subsequently dried by controlled low-temperature vacuum (CLTV) drying [57] (1–10 mbar, initial temperature 25 °C, 24 h). The fourth (still open) side was then heat-sealed as described above and the pouches were stored in a desiccator at room temperature (RT)–for details see Fig. S2 and reference [58]. Based on the bacterial cell count (CFU) added to the pouches, we calculated (from at least three independent experiments) the initial number of probiotic bacteria per cm^2^ of nonwoven insert to be as follows: *S. salivarius* K12: 0.06–0.25 × 10^8^ CFU/cm^2^; *S. salivarius* M18: 0.06–0.125 × 10^7^ CFU/cm^2^; *L. plantarum* 8P-A3: 0.18–0.25 × 10^10^ CFU/cm^2^.

To test the viability of the probiotics after drying, we counted those in microliter tubes after storage for 1 day, in the case of *S. salivarius* K12 also after 6 months. The survival rate in tubes (200 µL aliquots) after storage was determined as follows: The dried cells were rehydrated by addition of 200 µL ddH_2_O and incubated at RT for 1 h. The cell number of the rehydrated cells was determined by serial dilution assays. Dilutions of *S. salivarius* K12 and *L. plantarum* 8P-A3 were inoculated on BHI and MRS agar, respectively, and the plates were incubated anaerobically at 37 °C for 1–2 days until visible growth. Survival rate after drying and storage was calculated as follows: survival rate = (cell number after drying/cell number before drying) × 100.

In addition, the survival rate in the pads after 3 months of storage at room temperature was estimated in the following way: One side of the pad was cut and the nonwoven containing the dried cells was removed and inserted into an Erlenmeyer flask containing 3 mL of PBS, then shaken at room temperature for 1 h at 150 rpm. Dilution series were made from the flask and inoculated onto BHI agar (for *S. salivarius* K12 and M18) or onto MRS agar plates (for *L. plantarum* 8P-A3), respectively. Plates were incubated for 1 to 2 days resp. at 37 °C under anaerobic conditions. Cell numbers of the dilutions were determined and the number of surviving probiotics calculated.

#### Testing of the Probiotic Containing Pads for Antimicrobial Activity Against Target Bacteria

To test the inhibitory activity of the probiotic-containing pads, two approaches were adopted depending on the favorable growth atmosphere of the pathogen. The strict and the facultative anaerobic target bacteria *C. acnes* and *S. aureus*, respectively, were incorporated into WC and BHI agar medium, respectively (cell density: 10^3^ CFU/mL), as described above in the membrane test. The dried pads with enclosed bacteria were then applied onto the surface of the agar—with or without a drop (~ 50 µl) of a commercial sterile hy (containing modified starch, 85% water content, commercially available for wound treatment, Draco®, Dr. Ausbüttel & Co. GmbH, Dortmund, Germany) as an interface between the semipermeable membrane of the pad and the agar surface. *S. aureus* test plates were incubated anaerobically at 33 °C for 48 h, whereas the anaerobe *C. acnes* containing plates were incubated at 33 °C for 5 days.

For strict aerobic or other facultative anaerobic target bacteria, the pouch was first applied onto the agar medium and incubated at the same conditions mentioned above. The pad was then removed, and the pathogens (15 µL, 10^5^ CFU/mL) were streaked in two perpendicular lines across the agar surface, where the probiotic containing pouch had been applied, analogous to the deferred antagonism test described. Subsequently, these plates were incubated aerobically at 33 °C for 18 h. In addition to the three pathogens listed in Table 2, *L. plantarum* 8P-A3 pouches were also tested against all the strains listed in Table 3.

**Table 3:** Other bacteria tested for antimicrobial activity of *L. plantarum* 8P-A3 containing pads

Cell wall structure	Bacterial strain	Origin	Antibiotic resistances (other than intrinsic, EUCAST abbreviations [60])
Gram positive	*Cutibacterium acnes* AB 1,548,052 (clinical isolate)	University Hospital Aachen	None
	*Cutibacterium acnes* AB 1,548,053 (clinical isolate)	University Hospital Aachen	None
	*Cutibacterium acnes* AB 1,548,016 (clinical isolate)	University Hospital Aachen	None
	*Enterococcus faecalis* DSM-2570 = ATCC 29,212	DSMZ	None
	*Staphylococcus aureus* ATCC BAA-1717 / USA300 (MRSA)	ATCC	MEH
	*Staphylococcus aureus* (MSSA) AB 161 1075 (clinical isolate)	University Hospital Aachen	FUS
	*Staphylococcus aureus* (MSSA) AB 161 1506 (clinical isolate)	University Hospital Aachen	BEN
	*Staphylococcus aureus* (MRSA) AB 156 1008 (clinical isolate)	University Hospital Aachen	BEN, MEH, FUS
	*Staphylococcus aureus* (MRSA) AB 161 1512 (clinical isolate)	University Hospital Aachen	BEN, MEH, LEV, ERY, CLI
	*Staphylococcus aureus* (MRSA) AB 157 2004 (clinical isolate)	University Hospital Aachen	BEN, MEH, LEV, ERY, CLI
	*Staphylococcus epidermidis* DSM 1798	DSMZ	None
	*Lactobacillus plantarum* subsp. *argentoratensis* DSM-16365	DSMZ	Information not relevant^a^
Gram negative	*Klebsiella pneumoniae* DSM 26,371 = ATCC 700,603	DSMZ	ESBL due to SHV-18, CHL, TET
	*Acinetobacter baumannii* DSM 105,126 = ATCC 17,978	DSMZ	Genes for ESBL and a carbapenemase present^b^
	*Pseudomonas aeruginosa* AB 1711 102 (clinical isolate)	University Hospital Aachen	PIP, PIT, CIP
	*Pseudomonas aeruginosa* AB 172 1520 (clinical isolate)	University Hospital Aachen	PIP, PIT, CTZ, CEP, AZT
	*Pseudomonas aeruginosa* AB 172 1720 (clinical isolate)	University Hospital Aachen	IMI, CIP

^a^Strain only used for establishing the antimicrobial activity of *L. plantarum* 8P-A3 in acid environment — not considered as pathogen here

^b^*A. baumannii* DSM-105126 is not a recent clinical isolate (as those obtained from the Aachen University Hospital); the presence of an extended-spectrum beta-lactamase and a carbapenemase was derived from its whole genome (GenBank accession CP000521.1) with ResFinder [59]

#### Bacteriocin Production by *L. plantarum* 8P-A3 and Analysis

In order to prove that an identified inhibitory activity against target bacteria is resulting from antimicrobial peptides produced and to exclude that the inhibition is solely the result of lactic acid and low pH or other unspecific effects, experiments with culture supernatants of *L. plantarum* 8P-A3 were performed. Assuming that another *Lactobacillus* strain as indicator bacterium is not sensitive to lactic acid but may be sensitive to the bacteriocin(s) of *L. plantarum* 8P-A3, we selected the *L. plantarum* subsp. *argentoratensis* strain DSM-16365. This was based on the fact that the genome of strain DSM-16365 (GenBank accession no. CP032751) is lacking the bacteriocin locus present in the genome of *L. plantarum* 8P-A3 (Genbank acc. no. CP046726) [41]. This information was deduced from a nucleotide alignment of the bacteriocin locus of *L. plantarum* 8P-A3 to the chromosome of strain DSM-16365, using the alignment algorithm included in the Geneious Prime® bioinformatics software package (version 2020.1.2, https://www.geneious.com)—see Fig. S3 in the Supplementary Material.

In addition, to rule out the inhibitory effect of lactic acid in the following experiments, we used a test culture medium with a low glucose content. For this purpose, trypticase soy broth without dextrose (Becton, Dickinson and Company – BD Diagnostic Systems, Heidelberg, Germany) plus 0.5% yeast extract (Carl Roth GmbH & Co. KG, Karlsruhe, Germany) and 1.5% agar (TSAYE) was used. Another aspect was to rule out inhibition by hydrogen peroxide (H_2_O_2_) by incubating the test plates under anaerobic conditions [40].

In order to detect the bacteriocin production, a modified spot on the lawn test [33, 40] was used, where the producer strain is cultivated on solid medium; this was performed as follows: 15 µL spots of *L. plantarum* 8P-A3 suspension was applied on plates containing TSAYE medium without dextrose in three dilutions in PBS (3 × 10^9^ CFU/mL, 3 × 10^8^ CFU/mL, and 3 × 10^7^ CFU/mL); then, the plates were incubated anaerobically for 24 h at 30 °C. The next day, TSAYE medium containing 0.8% agar was tempered to 45 °C and seeded with the bacteriocin sensitive *L. plantarum* DSM-16365 to a final density of 10^3^ CFU/mL. The spotted plates were overlaid with 5 mL of the seeded TSAYE agar, cooled-down, and then incubated anaerobically for 48 h at 30 °C. Inhibition of the indicator strain was detected by clear zones around the spots of *L. plantarum* 8P-A3 (Fig. S1).

#### Identification of Antimicrobial Resistance Genes in the Genome of *L. plantarum* 8P-A3

To prove, that no acquired antimicrobial resistance of the *L. plantarum* 8P-A3 strain would impair its value to be developed further as a probiotic for human use, its genome was screened for resistance genes using the ResFinder webserver at the Center for Genomic Epidemiology, Technical University (DTU), Lyngby, Denmark [59].

## Results

### Antimicrobial Activity Against Bacterial Skin Pathogens

The conventional antagonism test methods line test, double layer agar test, membrane test, and deferred antagonism test were applied to elucidate the antimicrobial activity of the probiotics against selected human pathogens.

In the line test, *S. salivarius* M18, *S. salivarius* K12, and *L. plantarum* 8P-A3 demonstrated the ability to inhibit the growth of the three pathogenic bacterial species initially tested, since less or no colonies of these pathogens were observed on the line where the probiotic had been applied (for examples see Figs. S4, S5 and S6 in the Supplementary Material and Table 4).

**Table 4:** Summary of the results of conventional tests (line test, double layer agar test, membrane test, and deferred antagonism test)

Probiotic	*S. salivarius* M18			*S. salivarius* K12			*L. plantarum* 8P-A3		
Pathogen	*S. aureus*	*P. aeruginosa*	*C. acnes*	*S. aureus*	*P. aeruginosa*	*C. acnes*	*S. aureus*	*P. aeruginosa*	*C. acnes*
Line test	+	+	+	+	+	+	+	+	+
Double layer agar test	+	+	+	+	+	NT	+	+	NT
Membrane test	+	Pathogen not suitable for the test*	+	+	Pathogen not suitable for the test*	+	+	Pathogen not suitable for the test*	+
Deferred antagonism test**	+	+	+	**	**	**	**	**	+

*NT* not tested

*P. aeruginosa does not grow well neither incorporated into the agar nor under the anaerobic conditions used in this test, **Some deferred antagonism tests with *S. salivarius* K12 and *L. plantarum* 8P-A3 did not yield unequivocal results; therefore, results are not listed. Results of this test have been reported to be markedly media-dependent [61]

**Fig. 1:**
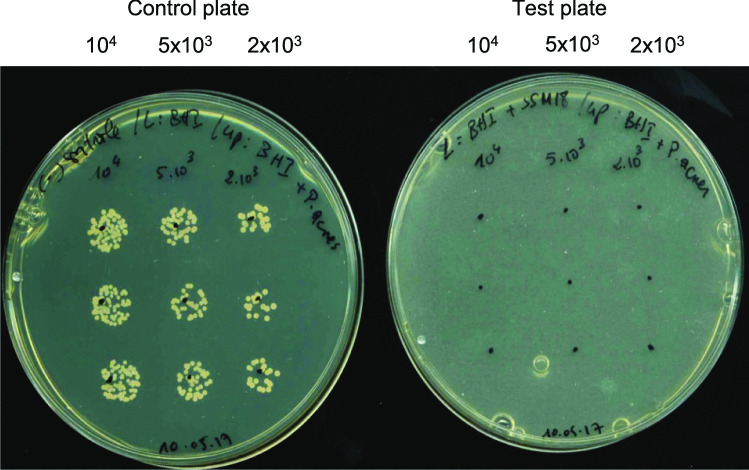
Double layer agar test with *S. salivarius* M18 against *C. acnes* DSM-1897

All the target strains tested for sensitivity to the three probiotics were unable to grow on the surface of the upper agar medium layer in the double layer agar test (Fig. 1 as an example for *S. salivarius* M18 against *C. acnes*). Compared to the control plate where the probiotics were absent in the lower agar medium layer, the growth of these pathogens was completely inhibited at all the inoculum densities applied (see also Fig. S7 and Table 4).

**Fig. 2:**
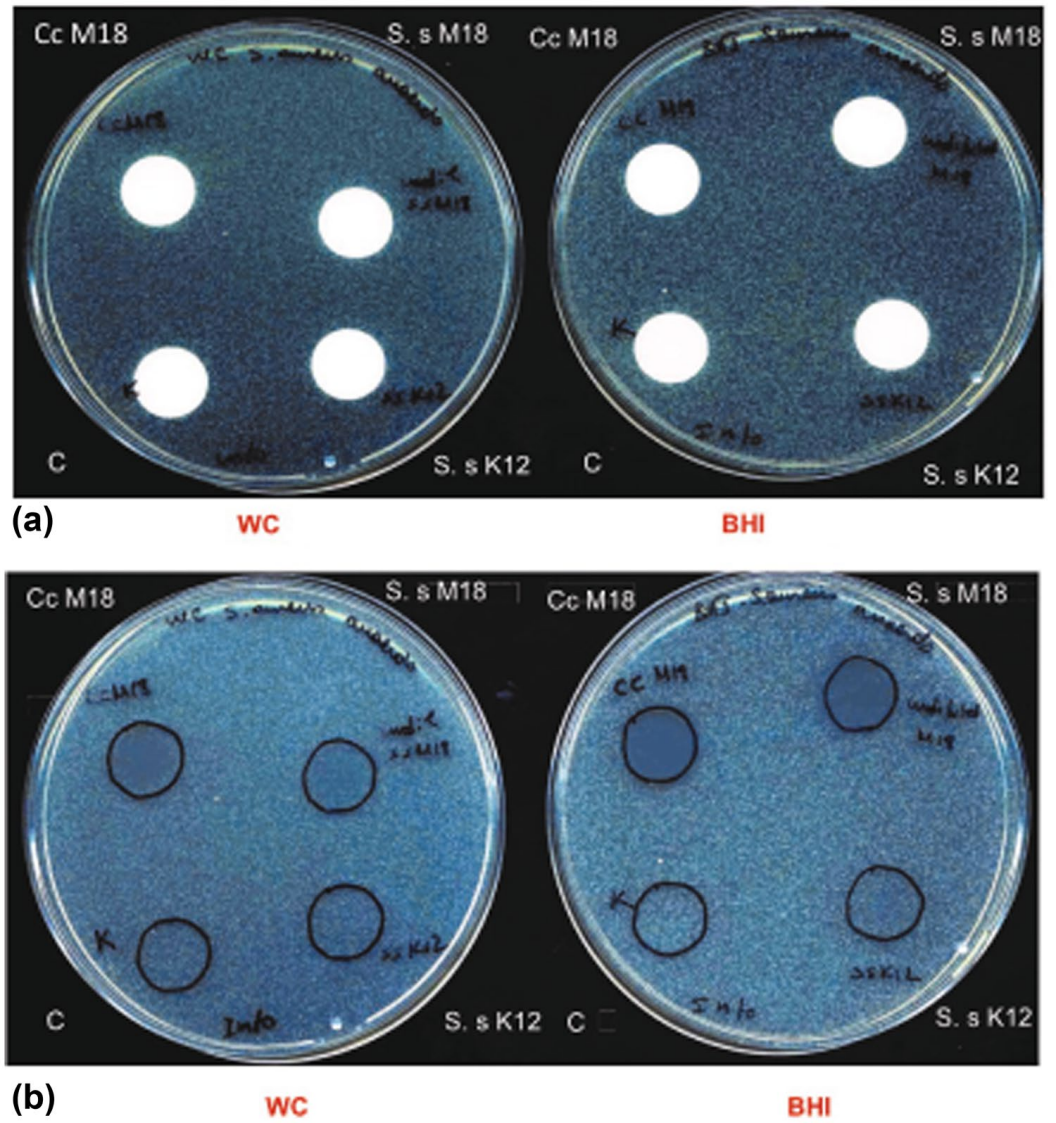
Membrane tests with *S. salivarius* M18 and K12 against incorporated *S. aureus* in WC and BHI agar medium: **a** Agar plates after incubation before removing the membranes; **b** Agar plates after incubation and removing the membranes. S. s M18: *S. salivarius* M18 (2 × 10^6^ CFU/mL), Cc M18: tenfold concentrated *S. salivarius* M18 (2 × 10^7^ CFU/mL), S.s K12: *S. salivarius* K12 (10^8^ CFU/mL); **c** Negative control (culture medium without probiotics)

The membrane test with *S. salivarius* M18 against the incorporated *S. aureus* showed that on BHI and on WC agar media, higher concentrations of the applied probiotic (tenfold concentrated, 2 × 10^7^ CFU/mL) resulted in reduced growth (fewer colonies) of the pathogen under the cellulose acetate membrane. These results could be more clearly observed after removing the membranes from the agar medium surface (Fig. 2). On the other hand, the BHI agar medium led to a better inhibition of *S. aureus* than WC medium with both cell densities of *S. salivarius* M18.

The antagonistic activity of *S. salivarius* M18 against *C. acnes* in the membrane test was more pronounced when the pathogen was incorporated into WC agar medium. This was easily observable even before removing the membranes from the surface of the agar medium, since the pathogen was inhibited not only under the membrane but also in the surrounding area, especially at higher cell counts of the probiotic.

*S. salivarius* K12 could not prevent the growth of *S. aureus* on both media in the experiment shown in Fig. 2; however, in a previous membrane test experiment (figure not shown), we had demonstrated inhibition of *S. aureus* also by *S. salivarius* K12. We also observed a clear inhibition when this probiotic was tested against *C. acnes*, comparable to that obtained with *S. salivarius* M18.

**Fig. 3:**
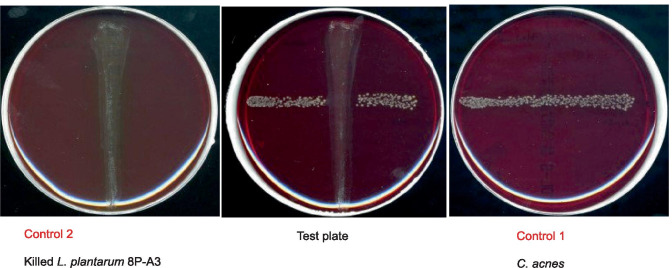
Deferred antagonism test with *L. plantarum* 8P-A3 against *C. acnes*. Control 1 without probiotic: *C. acnes* is able to grow on Columbia agar with 5% sheep blood. Control 2 verifies that the probiotic was completely killed after treatment with chloroform vapor

In the deferred antagonism tests, the plates displayed a clear absence of *C. acnes* colonies around the area, where the probiotics *S. salivarius* M18 and *L. plantarum* 8P-A3 (Fig. 3 and S8) had been applied.

The results of these conventional tests for antimicrobial activity of the probiotics applied are summarized in Table 4; the table includes additional test results not shown in the figures. In summary of these experiments, we state that the three probiotics are able to inhibit the growth of the three major skin pathogens tested.

### Probiotic Containing Pads with Antimicrobial Activity Against Potential Skin Pathogens

From the CLTV-drying experiments performed in tubes with 5% trehalose as protectant, the overall survival rate of *S. salivarius* K12 and *L. plantarum* 8P-A3 within the pads after drying can be calculated to be at least 30%, i.e., the number of viable bacteria (CFU) being more than 10^7^ per pad. For *S. salivarius* M18, this resulted in more than 10^6^ CFU/pad.

After having established that the selected probiotics clearly inhibit representatives of the three target pathogens in the screening methods (Figs. 1, 2, and 3 and S4 to S8), the newly designed probiotic-containing pads proved active against these potential skin pathogens inoculated into agar or inoculated onto agar surfaces respectively (Figs. 4a, b and S9a–e).

**Fig. 4:**
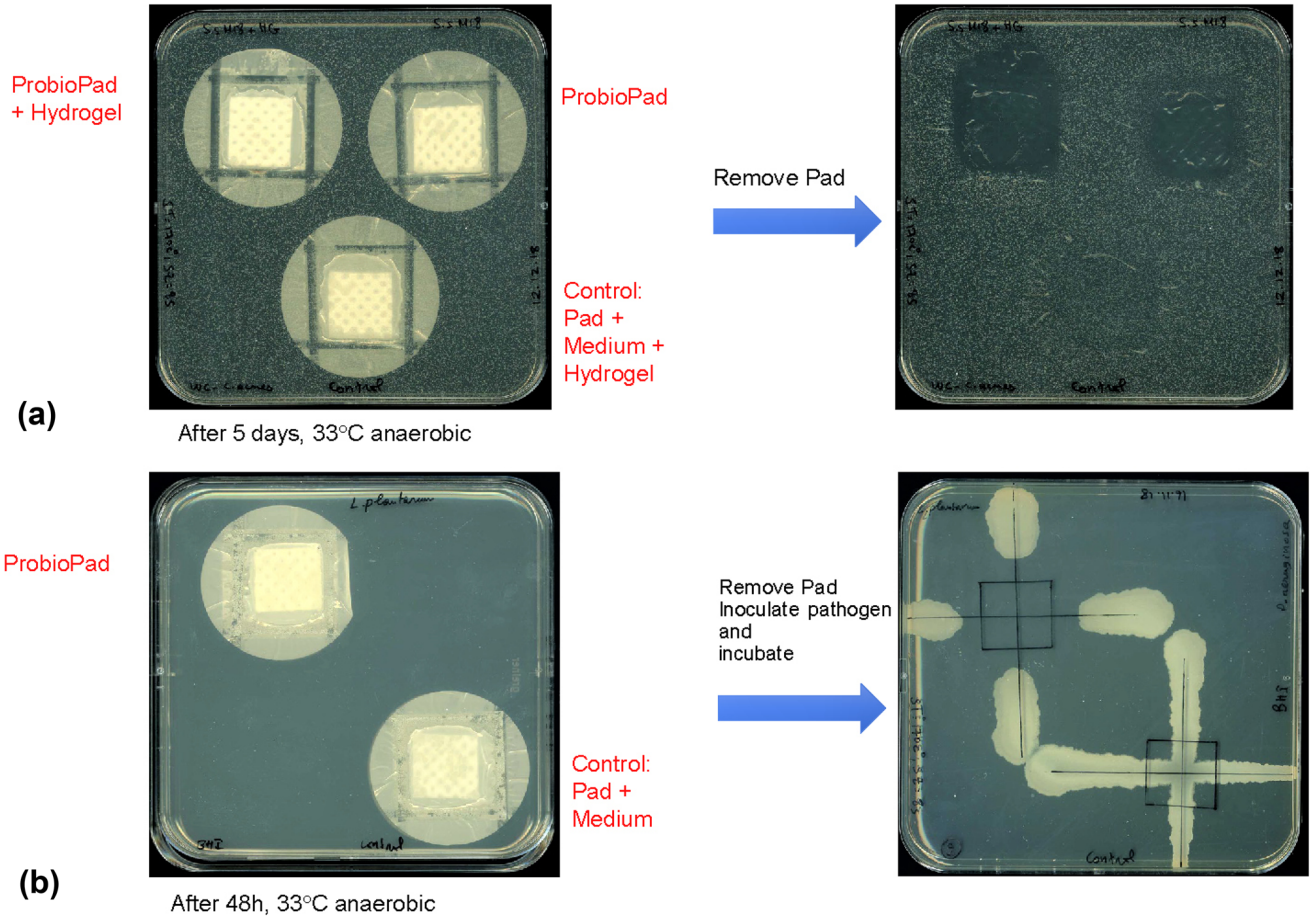
Test of the probiotics containing pads against skin pathogens: **a** *S. salivarius* M18 pad vs. *C. acnes* incorporated into the agar; after 5 days of anaerobic incubation, the pads were removed to visualize the inhibition of the pathogen; inhibition was also achieved when one drop of a commercial hydrogel (Draco®) had been placed between the pad and the agar surface; **b** *L. plantarum* 8P-A3 pad vs. *P. aeruginosa* inoculated onto the agar surface after removal of the pad; after further incubation, the inhibition of *P. aeruginosa* becomes clearly visible

Pads containing the three probiotic bacteria were able to exert an antimicrobial activity against the pathogens listed in Table 2; examples of these results are shown in Figs. 4a and b (further examples see Fig. S9a–e).

Clear zones in/on the agar medium observed in these experiments showed that antimicrobial substances produced by *S. salivarius* K12, *S. salivarius* M18, and *L. plantarum* 8P-A3 diffused through the semi-permeable membrane and a hydrogel layer and inhibited the growth of the pathogens. Moreover, applying the hydrogel under the control pad (without dried probiotics) did not result in any inhibition of the target bacteria. The hydrogel was applied here to simulate the projected application of the pads onto human skin where an additional water source may be necessary for an effective reactivation of the probiotic in the pad.

The following quantitative data on the viability of the dried probiotics after different times of storage were obtained for *S. salivarius* K12 after drying in tubes with 5% trehalose and storage in a desiccator at RT:

- Initial cell density of the suspension before the drying: 2.09 × 10^8^ CFU/mL,
- Cell count after drying and storage for one day: 1.27 × 10^8^ CFU/mL,
- Viability after six months storage: 7.9 × 10^6^ CFU/mL

Viability of *L. plantarum* 8P-A3 after drying in tubes with 5% trehalose and storage as above:

- Initial cell density of the suspension before the drying: 4,18 × 10^10^ CFU/mL,
- Cell count after drying and storage for 1 day: 1.88 × 10^8^ CFU/mL.
  Viability of *L. plantarum* 8P-A3 after drying in tubes with 25% sorbitol and storage as above:
- Initial cell density of the suspension before the drying: 3.05 × 10^10^ CFU/mL
- Cell count after drying and storage for 1 day: 1.87 × 10^10^ CFU/mL.

The viability of the probiotics inside the pads could also be estimated from residual pads available after three months of storage at RT by removing the nonwoven inlay, shaking it in PBS as described in Materials and Methods: From a pad with *L. plantarum* 8P-A3 with 25% of sorbitol as protectant a cell count of 7.32 × 10^5^/mL resulted (calculated on the basis of 250 µL of culture added to the pad originally). For a pad with *S. salivarius* K12 with 5% trehalose as protectant the corresponding number was 7.8 × 10^3^/mL.

Independently of these cell counts, after storage for 3 months in a desiccator at RT, the pads containing the dried probiotics could be reactivated and their inhibitory activity (tested against *S. aureus* ATCC 6538, *P. aeruginosa* DSM-1117, and *C. acnes* DSM-1897) was maintained (example see Fig. S9e).

The aerobic pathogens *P. aeruginosa* DSM 1117 and *P. aeruginosa* AB 172 1520 were tested against the probiotic *L. plantarum* 8P-A3 by a deferred test approach (Figs. 4b and S9c and d) comparable to the deferred antagonism test described above. Here we observed that the growth of the target bacteria was also inhibited on the agar surface, where the dried probiotic pad had been applied. This means that the antimicrobial producer strain was reactivated, and the inhibitory substances had diffused through the semi-permeable polycarbonate membrane.

Importantly, pads containing *L. plantarum* 8P-A3 were not only active against the pathogens initially tested (listed in Table 2) but also against all clinical isolates of the species *S. aureus*, *C. acnes*, and *P. aeruginosa* screened, including recent multi-resistant isolates (Table 3). Moreover, these pads were active against further collection strains of *S. aureus* (ATCC BAA-1717 = MRSA USA300), *S. epidermidis* (DSM-1798), *Enterococcus faecalis* DSM-2570, *Klebsiella pneumoniae* (DSM-26371), and *Acinetobacter baumannii* (DSM-105126).

Since it is generally accepted that probiotics used as food additives or applied in another way to animals or humans should not be resistant to clinically applied antibiotics [62, 63], screening of the whole genome of the *L. plantarum* 8P-A3 for known antibiotic resistance genes [59] proved that it does not contain gene(s)—even at a 70% ID threshold—for transferable antibiotic resistance; under this aspect, there would be no restrictions for the application of this probiotic on the human skin.

## Discussion

In the present study, three selected probiotics (*S. salivarius* K12, *S. salivarius* M18, and *Lactobacillus plantarum* 8P-A3) were tested for their antimicrobial activity against the common skin and wound pathogens *C. acnes, S. aureus*, and *P. aeruginosa*. Pads containing these probiotics in a dried state were constructed and tested for their antimicrobial activity after reactivation on agar surfaces as a substitute for infected skin as target. Additionally, *L. plantarum* 8P-A3 containing pads were tested against selected strains from culture collections and clinical isolates—some multi-resistant—of *Acinetobacter baumannii*, *C. acnes*, *Enterococcus faecalis*, *Klebsiella pneumoniae*, *P. aeruginosa*, *S. aureus*, and *S. epidermidis.*

The advantage of employing probiotics (or their products) being antimicrobially active even against multi-resistant pathogens may be further supported by the assumption that a loss of activity against the targeted pathogens is still an unlikely or rare event [44, 45], in contrast to the frequent development of resistances to antibiotics commonly used against skin pathogens.

In our approach to tackle the problems associated with topical or systemic antibiotic treatments of skin and superficial wound infections, we decided for the use of live probiotic bacteria enclosed in the dormant state within polymer membranes in such a way, that—after reactivation—their antimicrobial products could diffuse through a semipermeable membrane on the skin-directed side [58]. We had chosen this construct to avoid a direct contact of the skin with the probiotics and subsequent colonization with them, but still allowing their diffusible products to act on diseased skin or infected wounds. The rationale behind this is to avoid an entry of the probiotics as opportunistic pathogens into the bloodstream of potentially immunosuppressed persons [46–48, 64].

To gain information if the selected probiotic bacterial strains (*S. salivarius* K12, *S. salivarius* M18, and *L. plantarum* 8P-A3; Table 1) are able to inhibit potential skin pathogens, we first applied established methods to one representative strain each of *C. acnes*, *S. aureus*, and *P. aeruginosa* (Table 2). The good activities of *L. plantarum* 8P-A3 seen here against *S. aureus* and *P. aeruginosa* are well in accordance with the results previously obtained for these pathogens by Tsapieva et al. [41]. Antimicrobial activity of *S. salivarius* K12 against *S. aureus* had also been reported previously [38].

Pads containing the three probiotic bacteria were able to inhibit the growth of all target bacteria listed in Table 2 (Figs. 4a, b and S9a–e). Upon application of the pads to human skin, it may be necessary to provide an additional water source for reactivation of the dried bacteria within the pads. To simulate the conditions on human skin, we tested the pads on agar with a hydrogel as an interface between the pads and the agar surface. This hydrogel did not interfere with the antimicrobial activity of the pads (probiotic-free control pads stayed inactive; Figs. 4a and S9a, b, and e). Besides providing water, application of such a hydrogel between the pads and the skin might provide a more intense contact to the rough surfaces of the skin.

As expected, we could demonstrate that the pads had maintained their antagonistic activity—viz. could be reactivated—after storage for at least three months in a desiccator at room temperature (Fig. S9e). Using a deferred method for testing the pads against the aerobe *P. aeruginosa*, the facultative anaerobe *S. aureus*, and the anaerobe *C. acnes*, we could demonstrate that a direct contact with the target pathogens (or products thereof) is not essential for sufficient production of inhibitory substances by the probiotic LAB chosen here (examples in Figs. 3, 4b, S8, and S9c and d).

*L. plantarum* 8P-A3 was also able to inhibit the growth of additional strains of *S. aureus*, *C. acnes*, and *P. aeruginosa* including the clinical isolates from these species listed in Table 3. Moreover, pads containing this probiotic inhibited other Gram-positives such as *Staphylococcus epidermidis* and *Enterococcus faecalis* as well as the Gram-negatives *Klebsiella pneumoniae* and *Acinetobacter baumannii.* These results are in agreement with the previous publication of Tsapieva et al. [41]; according to their data and our results, *L. plantarum* 8P-A3 can be considered as suitable probiotic to be further developed against skin pathogens using the approach described here.

Using the *L. plantarum* subsp. *argentoratensis* DSM-16365 as target bacterium, we could prove that the antibacterial activity of the pads containing *L. plantarum* 8P-A3 is probably caused by bacteriocin(s) and in any case not exclusively based on the action of lactic acid and/or hydrogen peroxide. We concluded this from the fact that under the conditions selected, namely, use of a production medium with low glucose content, anaerobic atmosphere, and low sensitivity of this LAB indicator bacterium for lactic acid, cultures of *L. plantarum* 8P-A3 exerted clear inhibition in the modified spot-on-the-lawn test.

Thus, there is good indication that the activity of the *L. plantarum* 8P-A3 containing pads is—at least in part—due to the diffusion and action of one or more bacteriocins produced by this probiotic. From the published nucleotide sequences of the plantaricin (*pln*) locus [41] and the whole genome of *L. plantarum* 8P-A3 (GenBank Acc. Nos. HQ651181 and CP046726.1 resp.) and comparison with other published sequences from *L. plantarum* strains, one can deduce that the following class IIb bacteriocins might be produced by our selected producer strain *L. plantarum* 8P-A3: plantaricin EF [65] and/or plantaricin NC8α/β [66, 67]. This had already been assumed by Tsapieva et al. on the basis of the *pln* locus sequence they had described [41]. They also had found that the plantaricin locus in the genome of the *L. plantarum* strain J51 has a nearly complete identical nucleotide sequence [68]. In preliminary experiments—data not shown, we could detect bands of the expected molecular mass of those bacteriocins applying SDS-PAGE of a concentrated, antimicrobially active culture supernatant of *L. plantarum* 8P-A3.

The pads with the enclosed probiotic *L. plantarum* 8P-A3 can be considered as a safe potential device for treating bacteria-associated skin disorders, like *Acne vulgaris* or superinfected skin lesions, e.g., in atopic dermatitis. Also, the broad antibacterial spectrum including *S. aureus* and *P. aeruginosa* confirmed here that it might be useful for even treating chronic wounds associated with venous leg ulcers or burn wounds as described recently for a different *L. plantarum* strain [69]. The safety of the pads with enclosed *L. plantarum* 8P-A3 developed here is also based on the finding that no gene for an acquired antibiotic resistance is present in this probiotic and that a direct contact with the living probiotic bacteria is avoided—as discussed above.

In contrast to the use of extracts or supernatants from probiotic bacteria for treatment of skin disorders described in the literature [17, 19, 21, 22], our construct of pads might lead to an increased yield of active bacteriocins, since products or constituents of the target pathogens could diffuse into the pads and induce bacteriocin production by interference with the quorum sensing system of the probiotics [50, 51].

In addition to the antimicrobial effect of probiotics on skin pathogens, other beneficial effects on the skin microbiome and/or wound healing were described [22, 70] and could result from the application of the probiotic pads. Those could be triggered by known immunomodulatory effects of probiotics [71]. The secreted products may also have beneficial immunomodulatory effects on the skin or on wounds, which are commonly described for the oral route of application of some probiotic strains—for review see [72].

However, also unfavorable effects on the skin microbiome may be possible: inhibiting beneficial strains of *C. acnes* or of *S. epidermidis* may lead to perturbation of the skin microbiome and to an exacerbation of the disease intended to treat [35, 73–75].

In conclusion, we present in vitro data on the broad antimicrobial activity of selected probiotic lactic acid bacteria against common skin pathogens. Moreover, we report on design and testing of patches (bandages, pads or plasters) enclosing those probiotics and intended for topical treatment of skin disorders and infected wounds.

To develop these patches further for cosmetic or medical applications, we consider the following studies as essential:

- Isolation, purification, and characterization of the bacteriocins produced by the *L. plantarum* 8P-A3 strain;
- Tests for in vitro activity against additional *C. acnes* isolates, especially those isolated from acne lesions and belonging to established acne-associated clones [75–77];
- Application of the pads to *ex-vivo* human skin and analysis of the microbiome changes and associated immunological parameters;
- A phase I clinical study in humans.

Those studies are in progress/projected in our laboratory now.

## Supplementary Information

Below is the link to the electronic supplementary material.

Supplementary file1 (PDF 2.70 MB)

## Data Availability

All raw data for the statements presented here are available from the authors EH and RL at the DWI Leibniz-Institute in Aachen, Germany.
