# Supplementary material for: A New Approach to Harness Probiotics Against Common Bacterial Skin Pathogens: Towards Living Antimicrobials
Source: Probiotics Antimicrob Proteins. 2021 Apr 15;13(6):1557–71. doi: 10.1007/s12602-021-09783-7 (PMC8578138; doi:10.1007/s12602-021-09783-7)
Supplement: Supplementary file 1 — Supplementary file1 (PDF 2.70 MB) [file 12602_2021_9783_MOESM1_ESM.pdf]

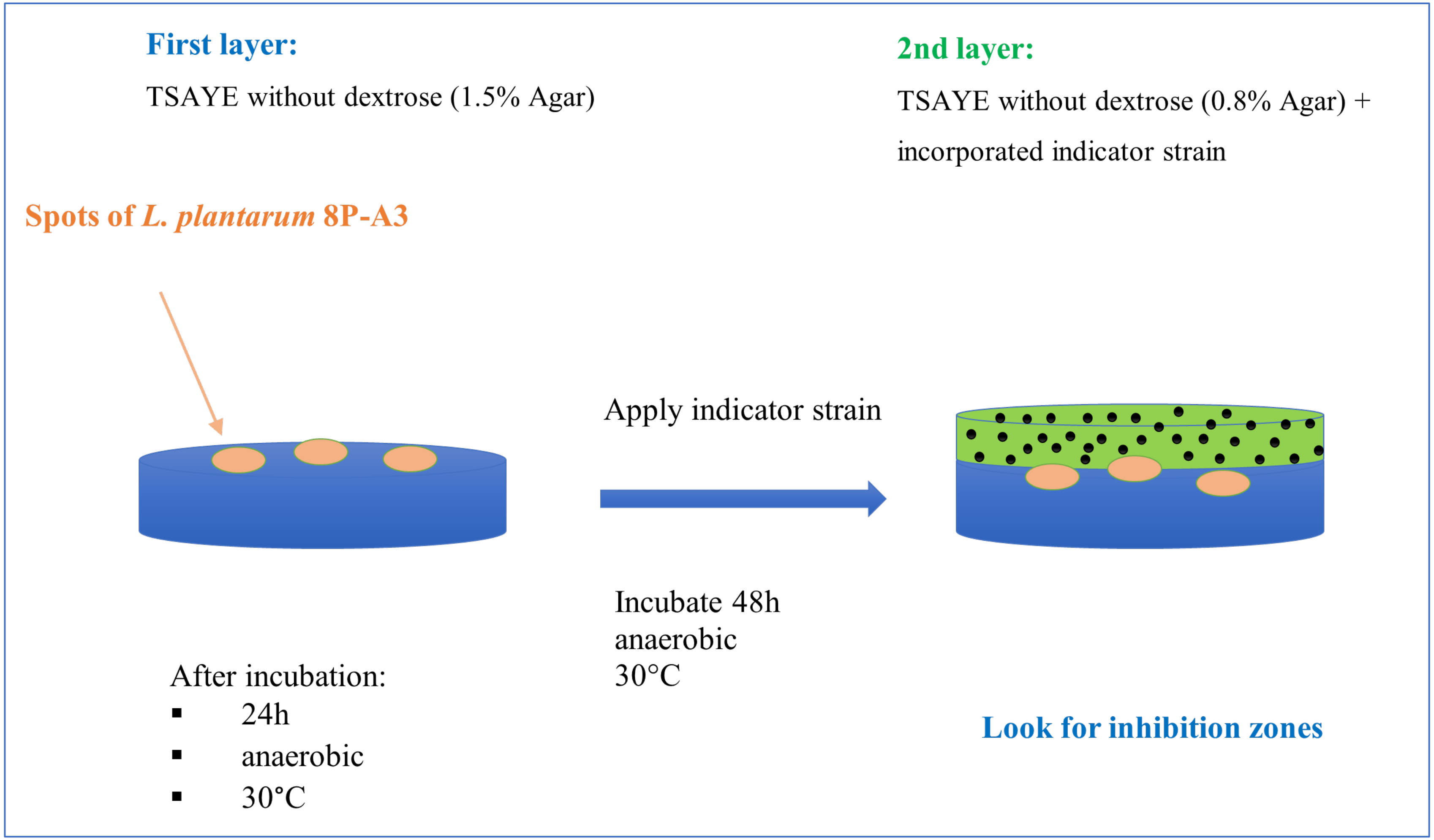

Fig S1 Scheme of the modified spot on the lawn test. Spots of the bacteriocin producer strain were applied onto the surface of the first layer (TSAYE with 1.5% Agar) and incubated anaerobically. After the appropriate incubation time, the indicator strain *L. plantarum* DSM-16365 was incorporated in TSAYE soft agar (0.8% agar) then applied on top of the first layer and incubated anaerobically for 48 h.

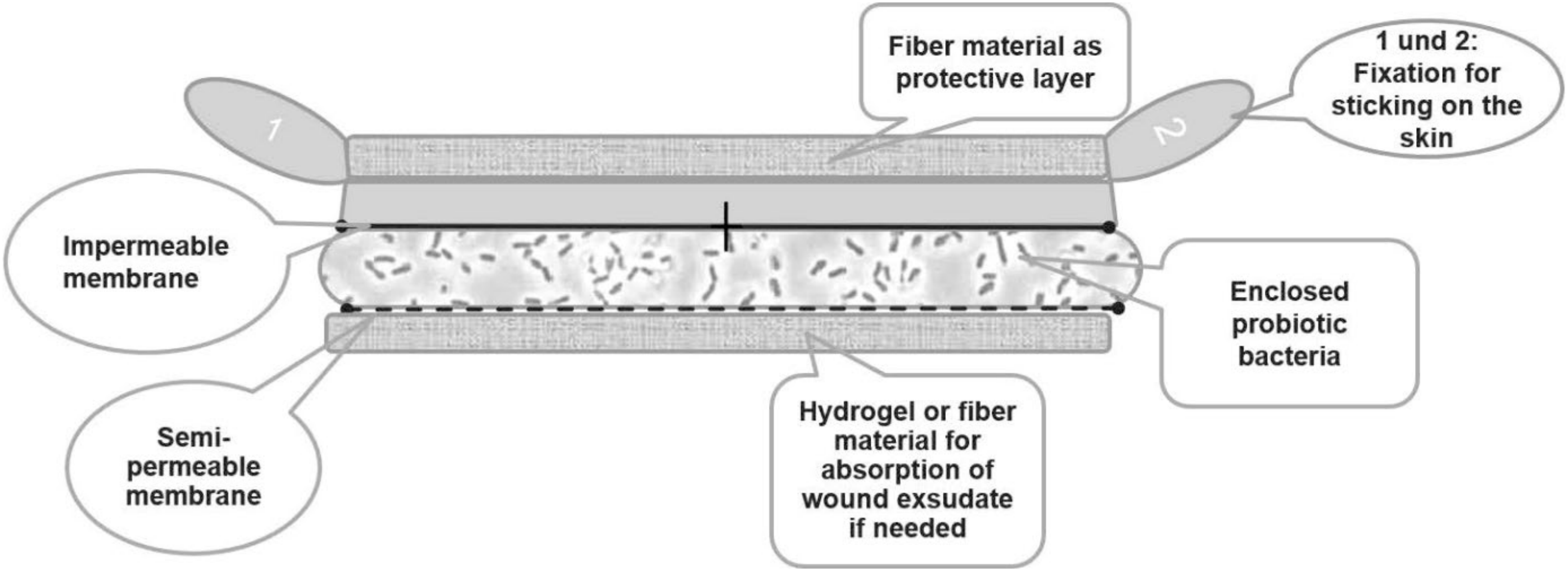

Fig S2 Schematic drawing of probiotic containing polymer pads. For details see Materials and Methods section and the respective patent application: Heine E, Lütticken R, Gartzen R, Khalfallah G, inventors; DWI - Leibniz-Institut für Interaktive Materialien e. V., 52074 Aachen (DE), assignee. Topical Formulation in Form of a Patch, a Bandage or a Plaster Comprising Probiotic Bacteria, and Use Thereof in a Method for Treating or Preventing Skin Disorders patent EP3366341A1. 2020 17.06.2020.

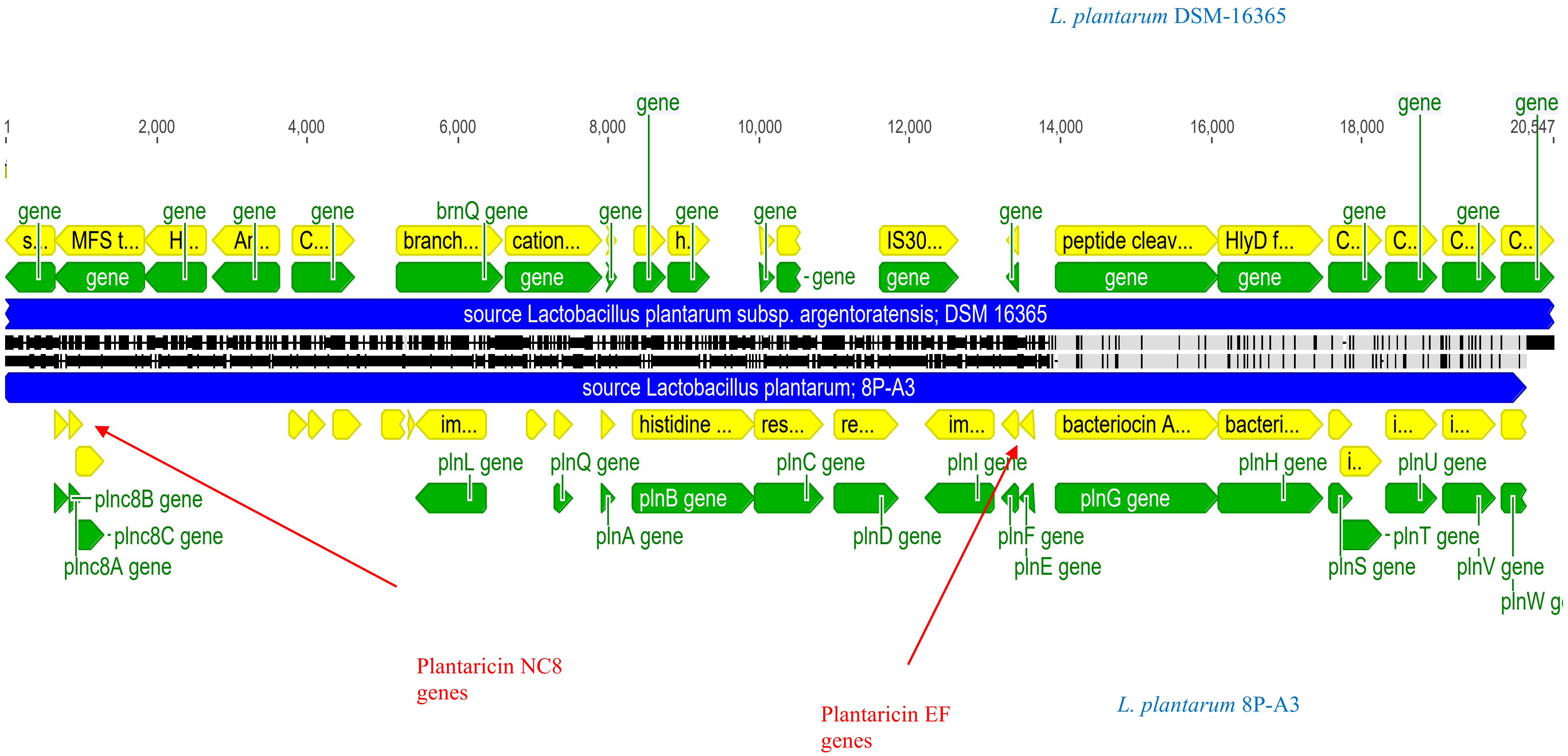

Figure S3 Nucleotide alignment of the plantaricin locus of *L. plantarum* 8P-A3 with the corresponding area on the *L. plantarum* DSM-16365 chromosome (generated with Geneious Prime® software suite)

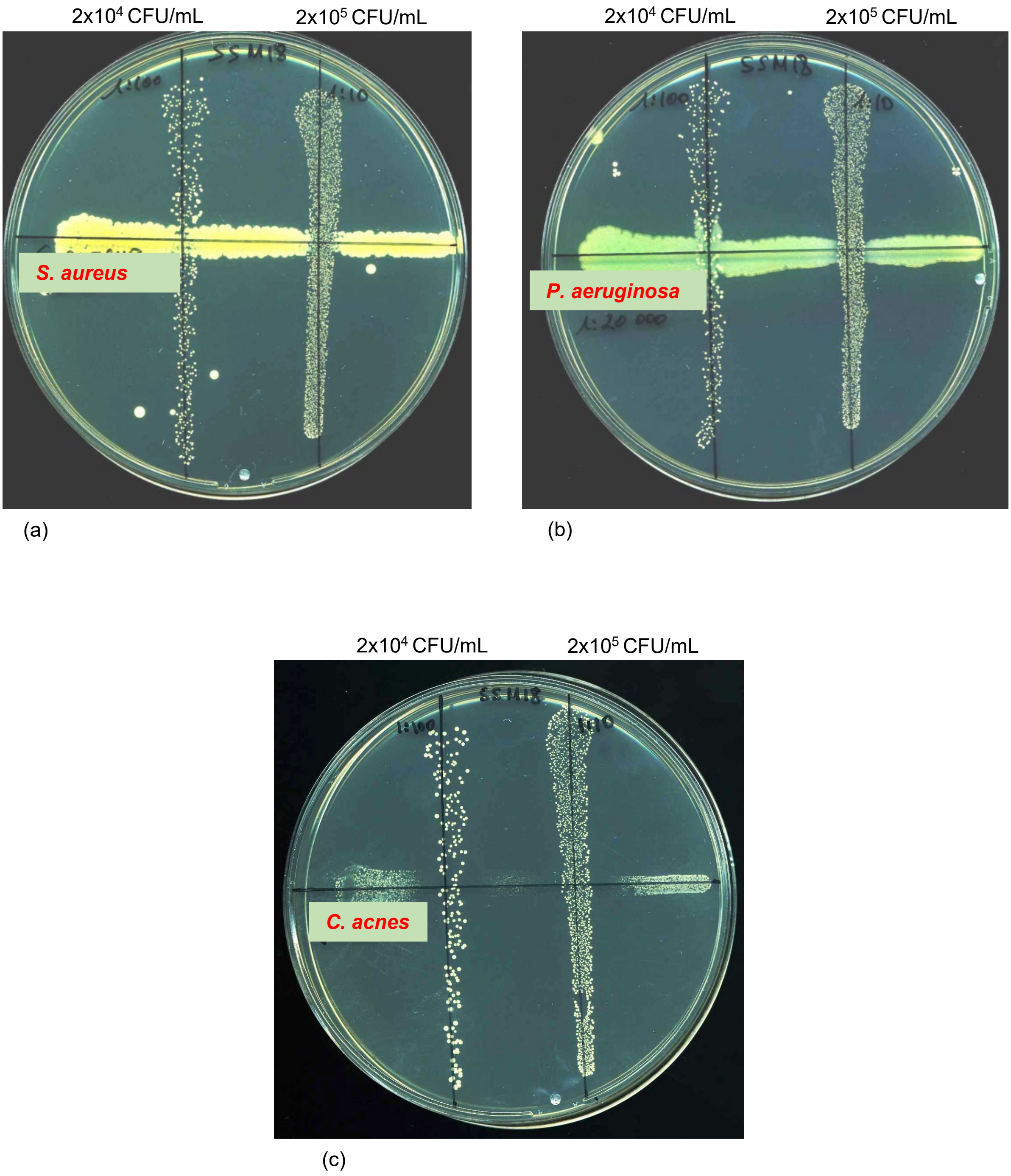

Fig S4 Line test with *S. salivarius* M18 against skin pathogens.: (a) *S. salivarius* M18 *vs. S. aureus,* (b) *S. salivarius* M18 *vs. P. aeruginosa* (c) *S. salivarius* M18 *vs. C. acnes.*

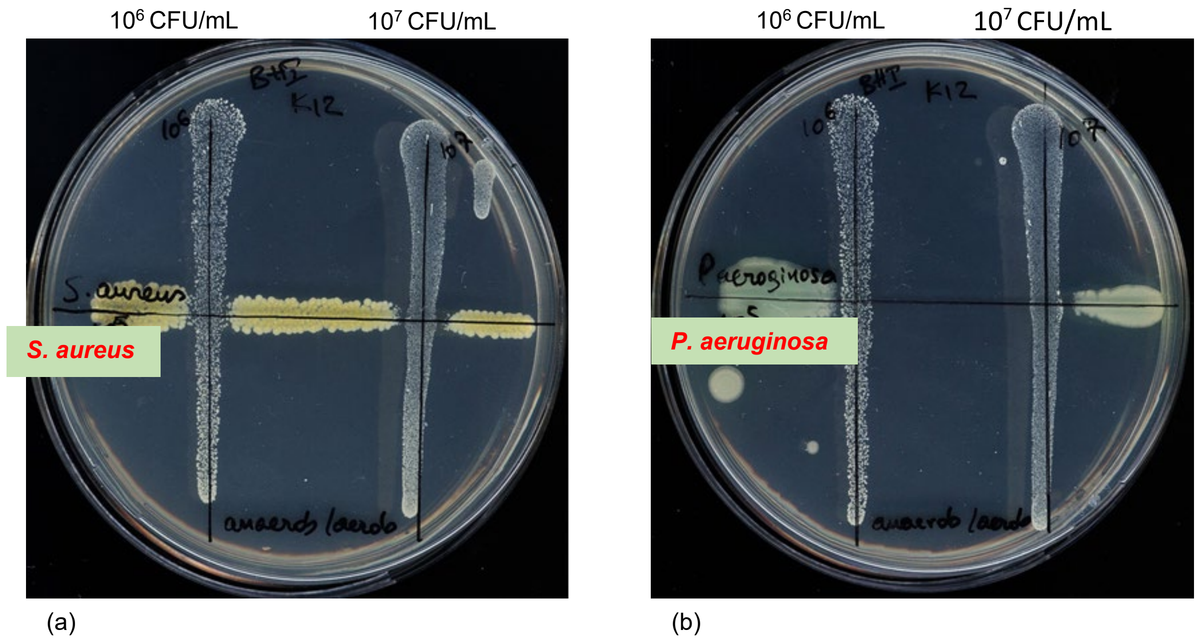

Fig S5 Line test with *S. salivarius* K12 against skin pathogens: (a) *S. salivarius* K12 *vs. S. aureus,* (b) *S. salivarius* K12 *vs. P. aeruginosa.*

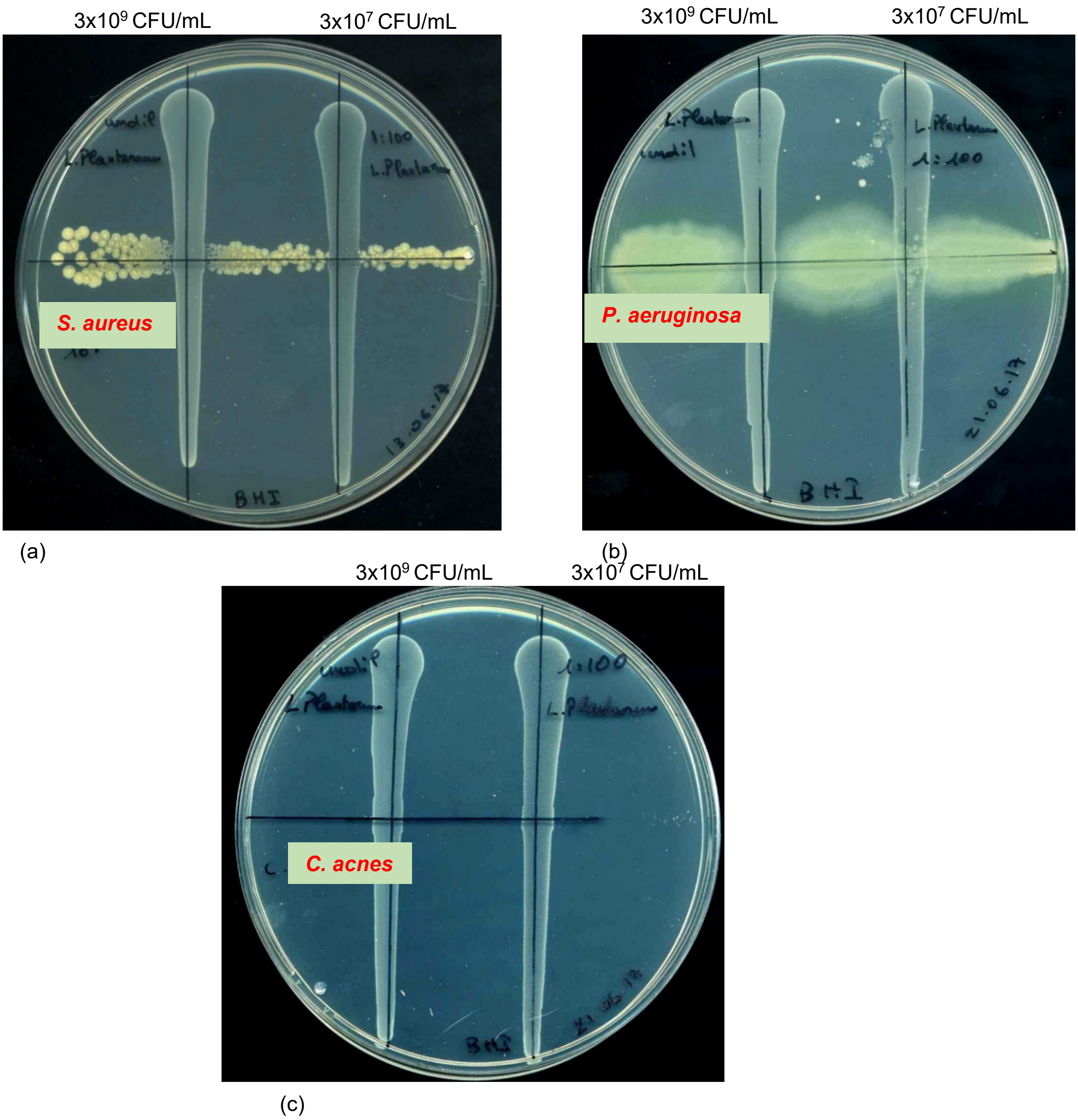

Fig S6 Line test with *L. plantarum* 8P-A3 against skin pathogens: (a) *L. plantarum* 8P-A3 vs. *S. aureus,* (b) *L. plantarum* 8P-A3 vs. *P. aeruginosa* (c) *L. plantarum* 8P-A3 vs. *C. acnes.*

The inhibition of *S. aureus* (Fig.1a, 2a, 3a) and *P. aeruginosa* (Fig.1b, 2b, 3b) was more remarkable when the probiotic had been inoculated with higher cell numbers. The growth of *C. acnes* (Fig.1c, 2c, 3c) was more affected by the presence of the three probiotics compared to the other indicator strains, as this pathogen could barely grow between the two probiotic streaks. The strongest growth inhibition of *C. acnes* was exerted by *L. plantarum* 8P-A3, since only few colonies on the margin of the plate were visible.

Control plate Test plate

10^4 $5x10^3$ $2x10^3$ 10^4 $5x10^3$ $2x10^3$

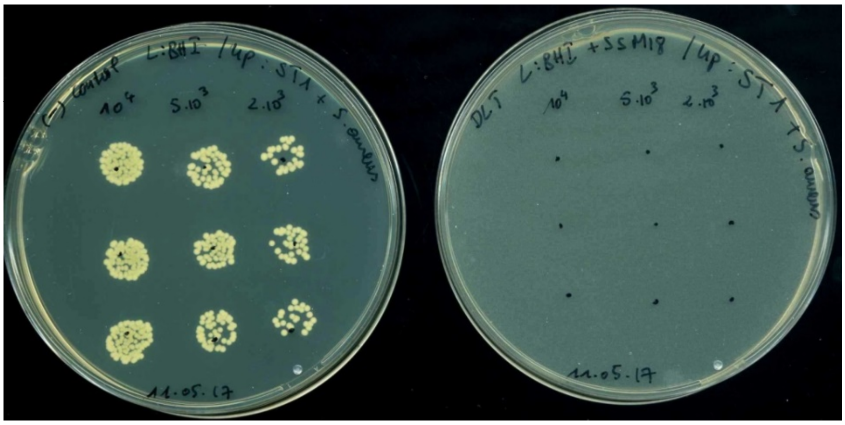

10^4 $5x10^3$ $2x10^3$ 10^4 $5x10^3$ $2x10^3$

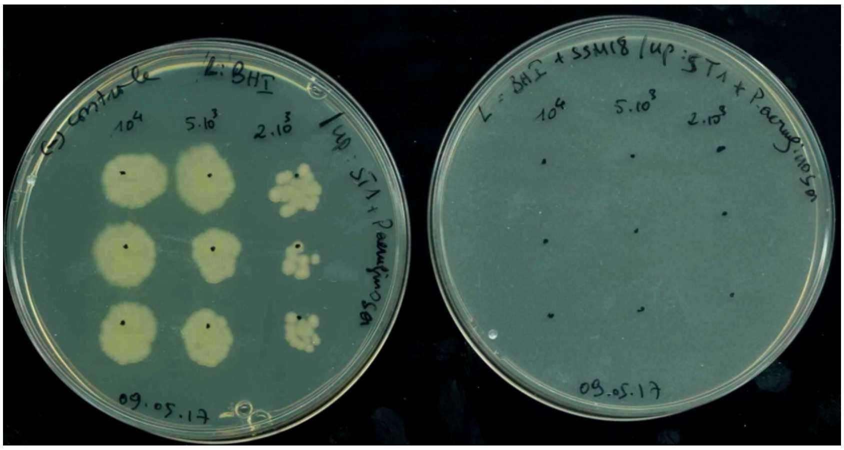

(b)

10^4 $5x10^3$ $2x10^3$ 10^4 $5x10^3$ $2x10^3$

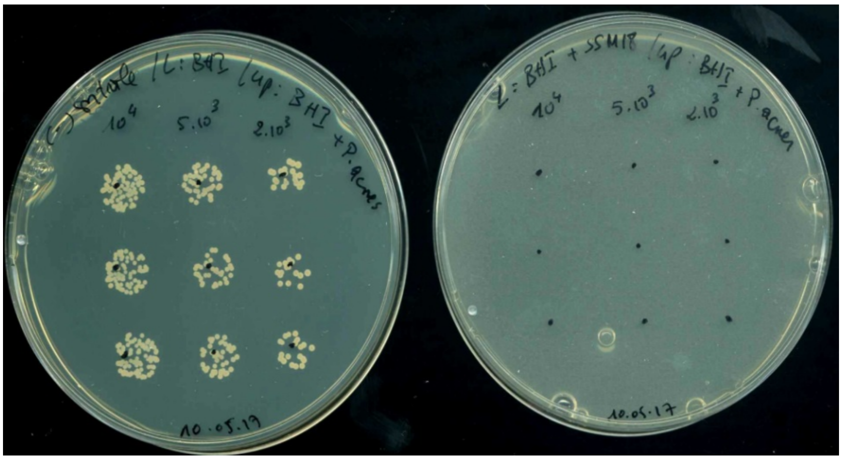

(c)

Fig S7 Double layer agar test with *S. salivarius* M18 against skin pathogens: (a) *S. salivarius* M18 *vs. S. aureus*. (b) *S. salivarius* M18 *vs. P. aeruginosa*. (c) *S. salivarius* M18 *vs. C. acnes*.

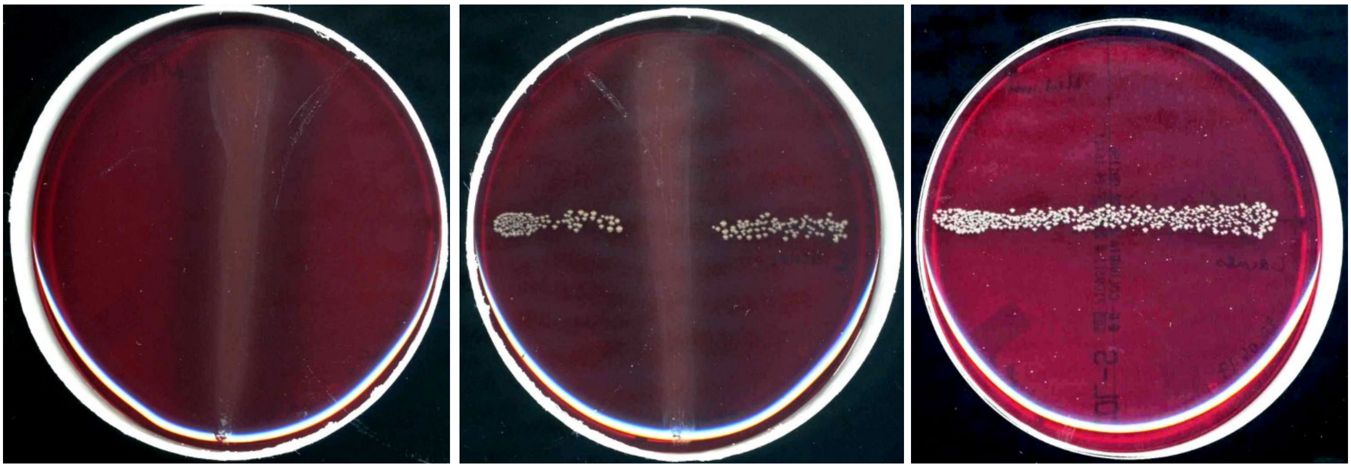

Control 2
Killed *S. salivarius* M18

Test plate

Control 1
C. acnes

(a)

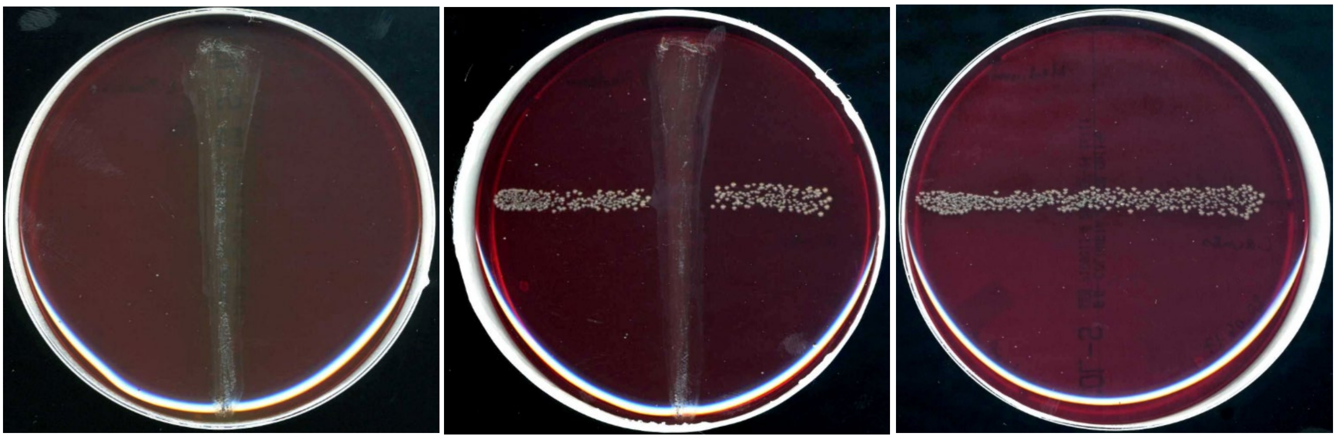

Control 2

Killed *L. plantarum* 8P-A3

Test plate

Control 1

C. acnes

(b)

Fig. S8 Deferred antagonism test. (a) *S. salivarius* M18 against *C. acnes*, (b) *L. plantarum* 8P-A3 against *C. acnes*

Fig. S9 Tests of the probiotics containing pads against pathogenic bacteria: (a) *S. salivarius* K12 pad vs. *S. aureus*, (b) *S. salivarius* M18 pad vs. *C. acnes* (c) *L. plantarum* 8P-A3 pad vs. *P. aeruginosa*, (d) *L. plantarum* 8P-A3 pad vs. *P. aeruginosa* AB 172 1520 (clinical isolate), (e) *S. salivarius* K12 pad vs. *S. aureus* after storage of the pads for 3 months.

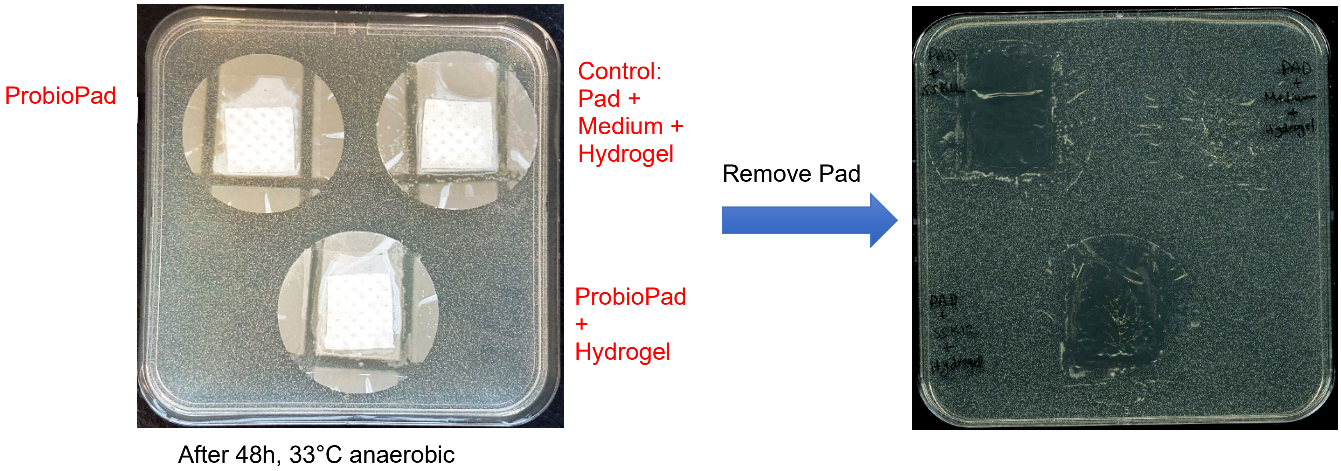

(a)

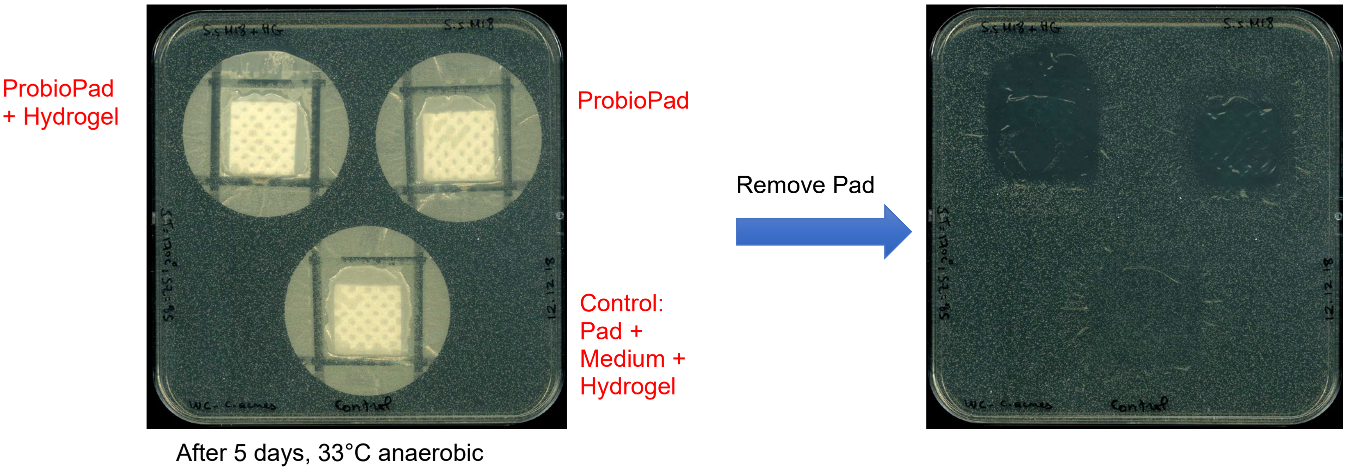

(b)

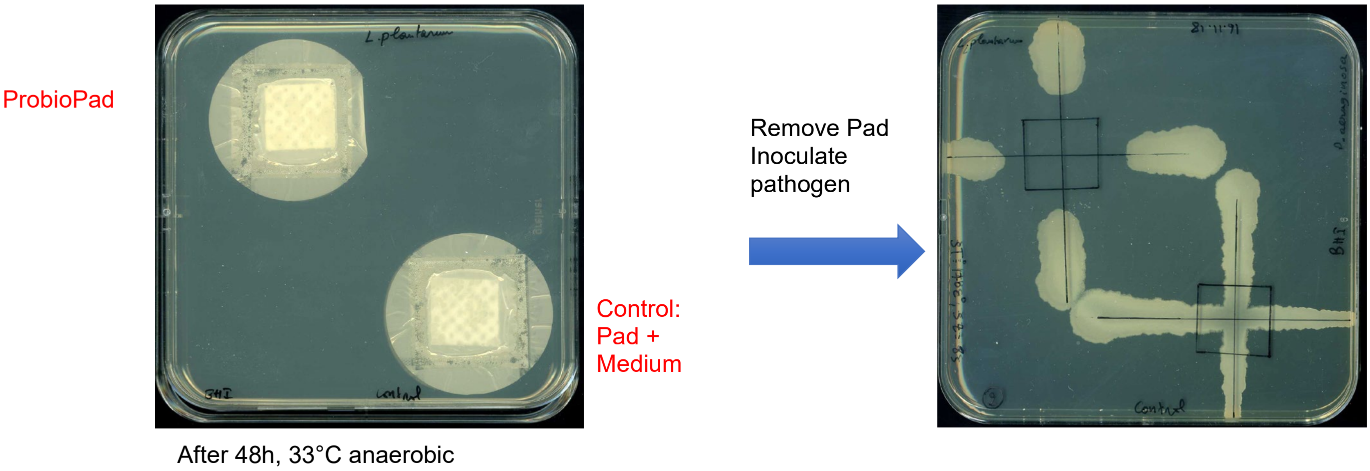

(c)

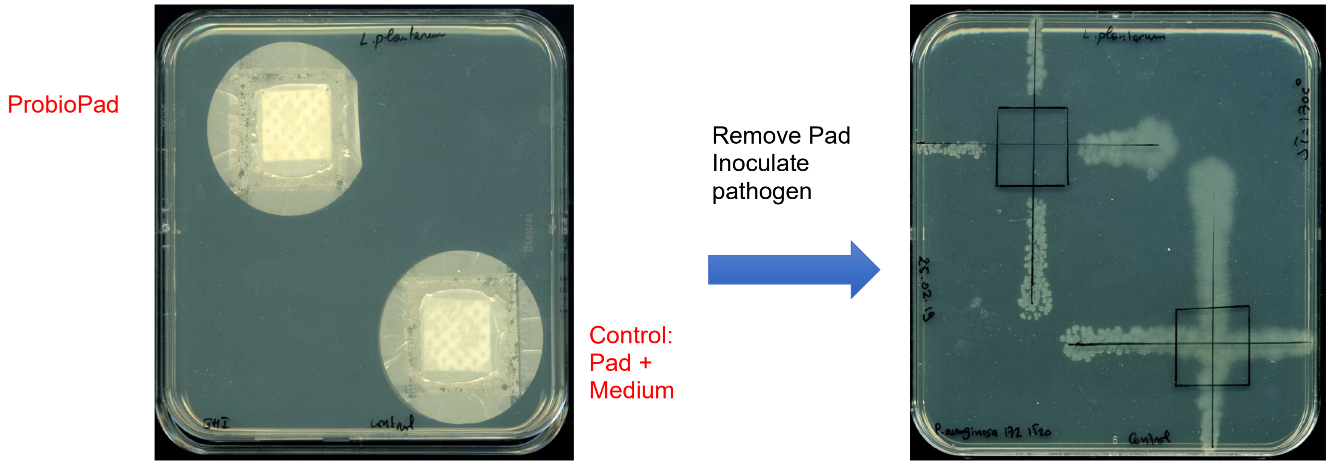

After 48h, 33°C anaerobic

(d)

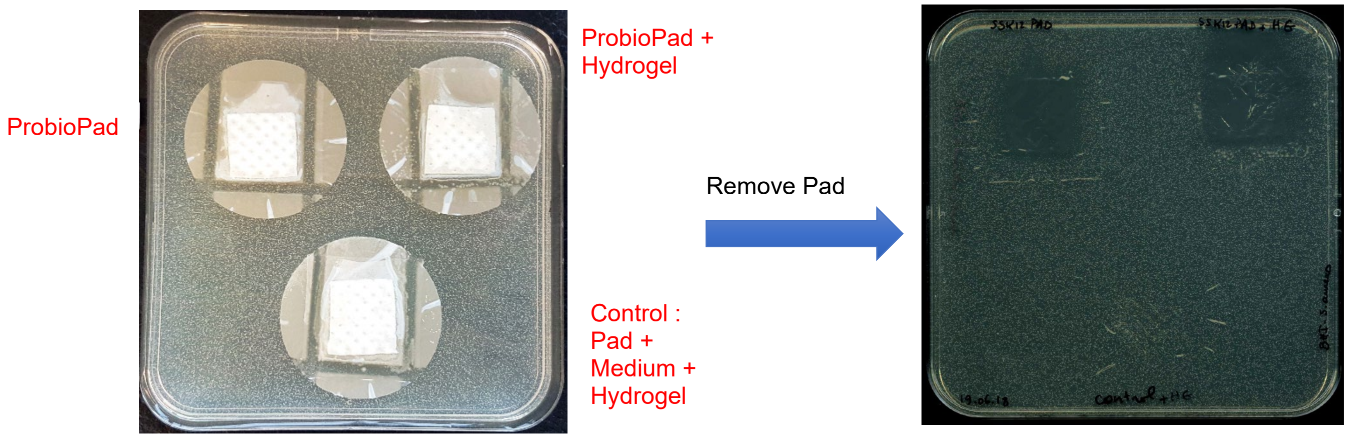

After 48h, 33°C anaerobic

(e)
